# *Plasmodium vivax* Populations Are More Genetically Diverse and Less Structured than Sympatric *Plasmodium falciparum* Populations

**DOI:** 10.1371/journal.pntd.0003634

**Published:** 2015-04-15

**Authors:** Charlie Jennison, Alicia Arnott, Natacha Tessier, Livingstone Tavul, Cristian Koepfli, Ingrid Felger, Peter M. Siba, John C. Reeder, Melanie Bahlo, Ivo Mueller, Alyssa E. Barry

**Affiliations:** 1 Division of Infection and Immunity, Walter and Eliza Hall Institute of Medical Research, Melbourne, Victoria, Australia; 2 Department of Medical Biology, University of Melbourne, Parkville, Victoria, Australia; 3 Papua New Guinea Institute for Medical Research, Goroka, Eastern Highlands Province, Papua New Guinea; 4 Swiss Tropical and Public Health Institute, Basel, Switzerland; 5 Centre for Population Health, Burnet Institute, Melbourne, Victoria, Australia; 6 Department of Mathematics and Statistics, University of Melbourne, Parkville, Victoria, Australia; 7 Barcelona Centre for International Health Research, Barcelona, Spain; University of Sao Paulo, BRAZIL

## Abstract

**Introduction:**

The human malaria parasite, *Plasmodium vivax*, is proving more difficult to control and eliminate than *Plasmodium falciparum* in areas of co-transmission. Comparisons of the genetic structure of sympatric parasite populations may provide insight into the mechanisms underlying the resilience of *P. vivax* and can help guide malaria control programs.

**Methodology/Principle findings:**

*P*. *vivax* isolates representing the parasite populations of four areas on the north coast of Papua New Guinea (PNG) were genotyped using microsatellite markers and compared with previously published microsatellite data from sympatric *P*. *falciparum* isolates. The genetic diversity of *P. vivax* (*H_e_* = 0.83–0.85) was higher than that of *P*. *falciparum* (*H_e_* = 0.64–0.77) in all four populations. Moderate levels of genetic differentiation were found between *P. falciparum* populations, even over relatively short distances (less than 50 km), with 21–28% private alleles and clear geospatial genetic clustering. Conversely, very low population differentiation was found between *P*. *vivax* catchments, with less than 5% private alleles and no genetic clustering observed. In addition, the effective population size of *P*. *vivax* (30353; 13043–69142) was larger than that of *P*. *falciparum* (18871; 8109–42986).

**Conclusions/Significance:**

Despite comparably high prevalence, *P*. *vivax* had higher diversity and a panmictic population structure compared to sympatric *P*. *falciparum* populations, which were fragmented into subpopulations. The results suggest that in comparison to *P*. *falciparum*, *P*. *vivax* has had a long-term large effective population size, consistent with more intense and stable transmission, and limited impact of past control and elimination efforts. This underlines suggestions that more intensive and sustained interventions will be needed to control and eventually eliminate *P*. *vivax*. This research clearly demonstrates how population genetic analyses can reveal deeper insight into transmission patterns than traditional surveillance methods.

## Author Summary

The neglected human malaria parasite *Plasmodium vivax* is responsible for a large proportion of the global malaria burden. Efforts to control malaria have revealed that *P*. *vivax* is more resilient than the other major human malaria parasite, *Plasmodium falciparum*. This study utilised population genetics to compare patterns of *P*. *vivax* and *P*. *falciparum* transmission in Papua New Guinea, a region where infection rates of the two species are similar. The results demonstrated that *P*. *vivax* populations are more genetically diverse than those of *P*. *falciparum* suggestive of a parasite population that is more resilient to environmental challenges, undergoing higher levels of interbreeding locally and between distant parasite populations. Unique characteristics of *P*. *vivax* such as relapse, which allows different strains from past infections to produce subsequent infections, may provide more opportunities for the exchange and dissemination of genetic material. The contrasting patterns observed for the two species may be the result of a differential impact of past elimination attempts and indicate that more rigorous interventions will be needed in efforts to control and eventually eliminate *P*. *vivax*.

## Introduction

*Plasmodium vivax* and *Plasmodium falciparum* are responsible for the majority of the human malaria burden worldwide. Malaria control and elimination initiatives have had enormous success, preventing an estimated 1.1 million deaths and approximately 274 million cases between 2001 and 2011 [1]. *P*. *falciparum* has traditionally attracted the greatest interest, as it is responsible for the majority of malaria deaths, while *P*. *vivax* has been relatively neglected. However, the classification of *P*. *vivax* malaria as “benign” has been revised in recent years as reports of severe vivax malaria have become commonplace in scientific literature [2,3]. Indeed, this species is estimated to be responsible for up to 300 million episodes of clinical malaria each year, predominantly in malaria-endemic regions outside sub-Saharan Africa [4]. Alongside the acknowledgment that *P*. *vivax* is of major global health significance, control programmes have revealed that this species is more resistant to control measures than *P*. *falciparum* [5]. Several unique features of *P*. *vivax* biology are thought to facilitate evasion of control efforts, including: relapse [6,7], the early appearance of transmission stages (gametocytes) [6,8] and a more rapid acquisition of clinical immunity [8,9]. *P*. *vivax* transmission is therefore likely to be more stable over time and during control efforts, when compared to *P*. *falciparum* [9].

Population genetic analyses using microsatellite markers have revealed important insights into malaria epidemiology, with genetically diverse populations suggesting endemic transmission [10–12] and clonal population structure signaling epidemic expansion [10–12]. The genetic diversity of *P*. *falciparum* populations is strongly associated with regional levels of transmission, thought to be the result of increasing proportions of multiple clone infections and consequent genetic recombination (outbreeding) in the mosquito vector [11,13,14]. *P*. *vivax* challenges this paradigm, maintaining high levels of diversity even in areas of low transmission [13–19]. Furthermore, *P*. *vivax* populations have consistently shown greater levels of genetic diversity than those of *P*. *falciparum* parasites circulating in the same region [15–19]. In South America, these differing patterns of diversity can primarily be explained by epidemic *P*. *falciparum* transmission leading to clonal expansion, whereas transmission of *P*. *vivax* is stable and hypoendemic [15,17]. In an area of South East Asia (Pursat, Cambodia), diversity of *P*. *vivax* was higher than *P*. *falciparum* despite a similar case prevalence (among all symptomatic malaria cases, 52% are caused by *P*. *falciparum*, 44% by *P*. *vivax* and, 4% by mixed infections) [17,20] however these numbers suggest higher *P*. *vivax* prevalence in the community since a lower proportion of *P*. *vivax* infections lead to clinical symptoms than *P*. *falciparum*. These findings raise important questions about how *P*. *vivax* diversity is generated and maintained, the relationship between diversity and endemicity and what consequences this knowledge may have for national control programs.

Genetic diversity secures evolutionary fitness, increasing the potential for adaptation to changing environments [20,21]. Indeed, genetically diverse parasite populations have greater potential to resist antimalarials [21–23], vaccines, and host immune responses [12,22–26]. Studies of the population structure of sympatric *P*. *falciparum* and *P*. *vivax* on local scales and at differing levels of transmission are needed to define potential drivers of genetic diversity in *P*. *vivax*. Population genetic studies are also necessary to guide malaria control and elimination strategies by tracing routes of transmission and the sources of epidemics [12,24–27], by identifying locations where the risk of reintroduction (gene flow) is lowest [27–30] and by monitoring drug and vaccine resistance [28–33].

On the north coast of Papua New Guinea (PNG) both *P*. *falciparum* and *P*. *vivax* are highly endemic, with *P*. *falciparum* entomological inoculation rates (EIR) marginally exceeding those for *P*. *vivax* [1,4,31–34]. In this region, the prevalence of *P*. *falciparum* rivals that of sub-Saharan Africa, while that of *P*. *vivax* is the highest in the world [1,4,34,35]. By comparing parasite population genetic structures, we aimed to gain an understanding of how local gene flow and genetic diversity differ between the two species in an area of similarly high prevalence. To investigate the genetic structure of sympatric *P*. *falciparum* and *P*. *vivax* populations, we determined multilocus microsatellite haplotypes in *P*. *vivax* isolates from four distinct geographic areas of PNG and compared the data to reanalysed, previously published data from the sympatric *P*. *falciparum* populations [35,36]. The results confirm the high diversity of these major malaria parasites in PNG and contrasting population genetic structures that highlight the potential consequences of the unique biology of *P*. *vivax* for malaria control programs.

## Methods

### Parasite isolates

Samples were collected from the Madang and East Sepik provinces on the north coast of PNG. In this region, all four major species of human malaria are endemic (*P*. *falciparum*, *P*. *vivax*, *Plasmodium malariae* and *Plasmodium ovale*), with year-round, intense transmission of malaria showing slight seasonal variations. In order to capture the diversity of parasites circulating in the community, venous blood samples were collected from 2359 asymptomatic volunteers of all ages in a cross sectional baseline survey at the start of an Intermittent Preventative Treatment of infants (IPTi) trial (Koepfli and Robinson *et al*. submitted). In the Wosera catchment area of the East Sepik Province, samples (n = 1077) were collected in the relatively dry period of August and September in 2005. The Wosera catchment comprises a cluster of eight villages spaced between 2–10km apart. In Madang Province samples were collected in the rainy season of March 2006 from twelve villages, clustered 5–20 km apart within three catchments areas surrounding Malala (n = 379), Mugil (n = 503) and Utu (n = 397) health centres, which are >50km apart (Table 1 and S1 Fig). These samples and the identification of parasite isolates have been described in detail elsewhere [35–37]. Only parasite isolates containing monoclonal infections were selected to ensure that multilocus haplotypes could be correctly reconstructed. Multiplicity of Infection (MOI) was previously determined using validated methods [37]. For the *P*. *falciparum* isolates, this included *Pfmsp2* [36–38] and for *P*. *vivax* isolates, *Pvmsp1*F3 and *PvMS16* genotyping [36,38–44].

**Table 1:** Prevalence and multiplicity of infection of *P*. *falciparum* and *P*. *vivax* populations on the north coast of Papua New Guinea.

Species	Province	Catchment	n	No. Infections (%)^a^	Infections with multiple clones (%)^b^	Mean MOI
*P*. *falciparum*	East Sepik	Wosera	1077	240 (22.3)	45	1.72
	Madang	Malala	379	129 (34.0)	39	1.59
	"	Mugil	503	195 (38.8)	44	2.01
	"	Utu	397	162 (40.8)	45	1.97
	TOTAL		2359	726 (30.8)	44	1.83
*P*. *vivax*	East Sepik	Wosera	1077	165 (15.3)	58	1.97
	Madang	Malala	379	131 (34.6)	48	1.80
	"	Mugil	503	167 (33.2)	48	1.76
	"	Utu	397	109 (27.5)	51	1.97
	TOTAL		2359	574 (24.3)	52	1.88

n = number of samples collected,

^a.^ based on LDR-FMA;

^b.^based on *Pfmsp2 or PvMS16/Pvmsp1f3; MOI = multiplicity of infection*

### Ethics statement

The samples were archived in a biobank at the PNG Institute of Medical Research. The original study in which samples were collected was explained in detail through both individual and community awareness meetings after which volunteers were invited to participate in the study. During enrolment, adult volunteers or the legal guardians of child volunteers were asked to provide oral informed consent to participate as this was the ethical requirement for this particular study, as approved by the local Institutional Review Board (details below). Whether oral consent was given to participate in the study and for samples to be used in further research, was documented in a database. Enrolment in the study was possible only if consent was given. All consenting members of selected populations were eligible for enrolment into the community surveys. People with concurrent or chronic illness that might impede their participation in the surveys were excluded. Ethical approval to conduct this study was granted by the PNG Institute of Medical Research Institutional Review Board (No. 11–05), the Medical Research Advisory Committee of PNG (No. 11–06) and the Walter and Eliza Hall Institute Human Research Ethics Committee (No. 11–12).

### Microsatellite genotyping

For *P*. *falciparum*, we used previously published data for 320 monoclonal *P*. *falciparum* isolates genotyped at ten previously validated and commonly used, putatively neutral, microsatellite markers including TA1, TAA60, Polya, ARA2, Pfg377, TAA87, TAA42, PfPK2, TAA81 and 2490 [35,45].

For *P*. *vivax*, eleven putatively neutral microsatellites were genotyped including; MS1, MS2, MS5, MS6, MS7, MS9, MS10, MS12, MS15, MS20 and Pv3.27, chosen as a result of their frequent use in other studies [39–44,46], thereby allowing our data to be compared with *P*. *vivax* data from previous studies [46]. The microsatellite markers were amplified using an 11-plex primary PCR followed by individual nested PCRs as previously described [35,46] with a total of 35 cycles were used in both the primary and secondary rounds of PCR. All PCR products were sent to a commercial facility for fragment analysis on an ABI3730xl platform (Applied Biosystems) using the size standard LIZ500.

### Data analysis

The *P*. *vivax* electropherograms were analysed with *Genemapper* V4.0 (Applied Biosystems) with the same peak calling strategy as that used for *P*. *falciparum* [35,47,48]. To avoid artefacts in the results that may occur with microsatellite markers [47–49] precautions were taken to ensure allele calling was as consistent as possible, including the reconstruction of dominant haplotypes (S1 Text). For both species the dominant and single haplotypes were compared within catchments to identify any significant differentiation by calculating both *G*_ST_ and Jost’s *D* in the *DEMEtics* R package (see below, [35,49]). The two datasets were pooled only if genetic differentiation was very low.

Previous analyses of the *P*. *falciparum* dataset identified strong to moderate population structure [35,50] and were based on diploid genotypes coded as homozygote at each locus. However, blood stage parasites are haploid and therefore both species were analysed here using haploid datasets, thus maintaining the correct sample size.

To identify outlier samples and markers, and as an alternative method for investigating population structure, multidimensional scaling (MDS) was performed on the haplotype datasets. MDS, an alternative to principal component analysis (PCA) that allows for missing data, aims to project the distance matrix of the data to a lower dimension *k*, while trying to minimise the distances between data points. MDS was performed with the set of dissimilarity measures (Euclidean distance). Multiple pairwise scatterplots of the transformed data were examined to determine whether sample outliers could be identified and clustering observed. These analyses were performed in the statistical software R [50,51], using the cmdscale function. PCA was also performed using the princomp R command. The biplot function in R plots the projection of the original microsatellite marker variables in the new data space was used to identify the outlier markers.

To conduct the population genetic analyses, allele frequencies and input files for the various population genetics programs were created using *CONVERT* version 1.31 [51,52]. Genetic diversity was measured by calculating the number of alleles (*A*) and expected heterozygosity (*H*_e_) using *ARLEQUIN* version 3.5.1.2 [52,53] and allelic richness (*R*_s_) using *FSTAT* version 2.9.3.2 [49,53]. Pairwise genetic differentiation was measured by calculating Jost’s *D* and the *F*_ST_-derivative, *G*_ST_ with bias correction using the *DEMEtics* package [49,54–56]. It should be noted that *G*_ST_ and its relatives have been shown to underestimate genetic differentiation when applied to diverse microsatellite markers [54–56]. Jost’s *D* however, is a more appropriate measure of genetic differentiation for diverse microsatellites, as it first normalises heterozygosity, thus allowing comparisons between species with different sets of microsatellite markers. Furthermore, Jost’s *D* is considered a superior diversity measure over *G*_ST_ and *F*_ST_ since it shows correct behaviour for highly polymorphic loci where *G*_ST_ and *F*_ST_ underestimate diversity [49,54,55,57]. We have therefore used Jost’s *D* as the primary measure of differentiation, however we have included *G*_ST_ to allow for comparison with previous studies. All markers (except TAA42 and Pv3.27, see results below) were used in these analyses regardless of their mode of mutation (simple step-wise or complex mutation). Jost’s *D* was calculated as follows:

$$D=\left[\left(H_{\text{T}}-H_{\text{S}}\right)/\left(1-H_{\text{S}}\right)\right]\;\left[n/\left(n-1\right)\right]$$

The bias-corrected *D*_est_ values were based on equation twelve of Jost 2008 [54]:

$$D_{\text{est}}=\left[\left(H_{\text{T}\_\text{est}}-H_{\text{S}\_\text{est}}\right)/\left(1-H_{\text{S}\_\text{est}}\right)\right]\;\left[n/\left(n-1\right)\right]$$

and *G*_ST_:

$$G_{\text{ST}}=\;\left(H_{\text{T}}-H_{\text{S}}\right)/H_{\text{T}}$$

Bias-corrected *G*_ST_est_ values were calculated according to Nei & Chesser [58]:

$$G_{\text{ST}\_\text{est}}=\left[\left(H_{\text{T}\_\text{est}}-H_{\text{S}\_\text{est}}\right)/H_{\text{T}\_\text{est}}\right]\;\left[n/\left(n-1\right)\right]$$

A Mantel test was performed between Jost’s *D* and *G*_*ST*_ and geographical distance between catchments, using the mantel.rtest function from the ade4 library in R, with 10,000 replicates [59]. Correlations between; prevalence, mean MOI and percentage of multiple infections with; *D* and *G*_*ST*_ were tested by measuring Spearman’s correlation coefficient (ρ).

To calculate effective population size (*N*_e_), the same method previously described for *P*. *falciparum* was used for both species [11]. Data for the mutation rate of *P*. *vivax* microsatellites are lacking, and therefore the microsatellite mutation rate (μ) for *P*. *falciparum* of 1.59×10–4 (95% confidence interval: 6.98×10^−5^, 3.7×10^−4^), was used for both species [46]. Not all markers adhere to a strict stepwise mutation model (SMM), therefore *N*_e_ was calculated using both the SMM and infinite allele models (IAM) [11]. For SMM, *N*_e_ was calculated as follows:

$$N_{e}\mu =\frac{1}{8}\left\{{\left[\frac{1}{1-H_{E}\_mean}\right]}^{2}-1\right\}$$

where *H*_E__*mean* is the expected heterozygosity across all loci. For the IAM, *N*_e_ was calculated using the formula:

$$N_{e}\mu =\frac{H_{E}\_mean}{4\left(1-H_{E}\_mean\right)}$$

As a measure of inbreeding in each population, multilocus linkage disequilibrium (LD) was calculated using LIAN version 3.6, applying a Monte Carlo test with 100,000 re-sampling steps [60]. The markers Pv3.27 (Pv) and TAA42 (Pf) and the sample outliers identified using the PCA biplot analysis were not included in LD analysis. To estimate associations among loci using this program, the Index of Association (*I* ^*S*^_*A*_) was calculated for all complete haplotypes and also those from single infections only. *I* ^*S*^_*A*_ was also calculated in single infections alone as a precaution against the potential for incorrectly reconstructed dominant haplotypes to artificially inflate outbreeding.

To further investigate parasite population genetic structure, the Bayesian clustering software, *STRUCTURE* version 2.3.4 was used to investigate whether haplotypes clustered according to geographical origin. Unlike the genetic differentiation parameters described above which are based on predefined populations, this program attempts to form groups of haplotypes based on the allele frequencies at each locus with no prior geographical information, assigning individuals to one or more populations (*K*) [61]. The analysis was run 20 times for *K* = 1 to 8 for 100,000 Monte Carlo Markov Chain (MCMC) iterations after a burn-in period of 10,000 using the admixture model and correlated allele frequencies. The log probability of the data *LnP[D]* used for determining optimal *K* has been shown to be suboptimal in some situations and therefore the second order rate of change of *LnP[D]*, *ΔK* was also calculated according to the method of Evanno *et al*. [62].

Due to the small number of data points (four catchments), statistical analysis of the resulting molecular epidemiological and population genetic parameters was done using non-parametric methods as indicated using R or *Prism* software (*GraphPad Prism*, version 6.0d, GraphPad software, San Diego) [50].

## Results

### Prevalence and MOI

As previously reported, amongst the 2359 blood samples collected, a total of 765 (30.8%) *P*. *falciparum* and 574 (24.3%) *P*. *vivax* infections were detected by molecular diagnostic methods [35–37]. The prevalence of both species was lowest in the Wosera and *P*. *falciparum* was the dominant species in all but one catchment (Malala), where prevalence was comparable (Table 1). The difference in species prevalence in the different catchments was not significant (two sample Mann-Whitney U test: *p* = 0.34). Based on genotyping using *Pfmsp2* and the combination of *Pvmsp1F3* and *PvMS16*, the mean MOI was only slightly greater for *P*. *vivax* (1.88) than that for *P*. *falciparum* (1.83, [37]) however *P*. *vivax* had a significantly larger proportion of multiple infections (52%) than did *P*. *falciparum* (44%) (Chi-squared test, 1 df: *p* = 0.0045).

### Identification of haplotypes and data cleaning

For *P*. *falciparum*, multilocus haplotypes with at least four of the ten microsatellites were available for 320 isolates including 214 confirmed single infections (single) and 106 “dominant” infections comprising dominant allele calls (major peaks) from two or more markers (dominant) [35] (S1 Dataset). Reanalysis of the cleaned dataset for the two groups of haplotypes again showed no genetic differentiation (S1 Table), and therefore the single infection and dominant infection datasets were combined for further analyses.

For the new *P*. *vivax* microsatellite data produced in this study, haplotypes for five or more microsatellites were successfully reconstructed for 204 *P*. *vivax* isolates [36]. Of these, 82 were single and 122 were dominant (S1 Dataset). Comparisons revealed negligible genetic differentiation, with all *G*_ST_ and *D* values being insignificant (S1 Table), therefore they were also combined for further analyses.

Before investigating population structure, the datasets were first screened using MDS and PCA to identify outlier markers or samples that might obscure signals of local population structure. For *P*. *falciparum*, this analysis identified 12 outlier samples that when removed, revealed separation between Madang and East Sepik samples and tight clustering of Utu samples within the Madang cluster (S2A Fig and S2B Fig). As indicated in the biplot for the PCA, the vertically aligned clustering pattern was driven by marker TAA42 (S2C Fig). This marker features a 57 bp indel and displayed a bimodal allele frequency distribution [35], which could either be due to the indel itself or due to genotyping artefacts. TAA42 was therefore removed from the *P*. *falciparum* dataset (S1 Dataset). For *P*. *vivax*, the analysis identified 11 outlier samples (S3A Fig). Furthermore, Pv3.27 introduced a clustering pattern independent of geographical origin (S3B Fig and S3C Fig). This marker was previously found to display excess diversity [63], which also occurs in these PNG populations. Pv3.27, as well as the 11 outlier samples, were consequently removed from all further analysis of *P*. *vivax* (S3 Table). Population structure is analysed in more detail below using the cleaned datasets.

### Genetic diversity

Levels of genetic diversity were high for both species in all four sympatric populations and all haplotypes were unique (Table 2). Genetic diversity was consistently higher for *P*. *vivax* for all parameters (Table 2). For *P*. *vivax*, estimates of genetic diversity within catchments were similar to that of all catchments combined, consistent with little or no population structure (Table 2). In contrast, the overall genetic diversity for *P*. *falciparum* was higher than that for the catchments (Table 2) consistent with a hierarchical level of population structure. For both species, Utu was the least diverse population, and all of the other catchments had similar levels of diversity (Table 2). No correlation was found between prevalence, mean MOI or percent of multiple infections with the levels of genetic diversity (S2 Table) indicating that diversity was not reduced as a result of lower transmission in the Wosera catchment, an observation we have previously made using other highly polymorphic markers [36,37].

**Table 2:** Estimates of genetic diversity of *P*. *falciparum* and *P*. *vivax* populations on the north coast of Papua New Guinea.

Species	Province	Catchment	*n*	*H*_*e*_ *±SE*	*A ±SE*	*R*_*s*_ *±SE*
						
*P*. *falciparum*	East Sepik	Wosera	110	0.74 ± 0.05	10.44 ± 0.96	9.33 ± 0.79
	Madang	Malala	62	0.77 ± 0.02	8.89 ± 0.81	8.64 ± 0.78
	"	Mugil	72	0.77 ± 0.03	9.33 ± 0.67	9.04 ± 0.69
	"	Utu	64	0.68 ± 0.06	7.78 ± 0.91	7.49 ± 0.87
	TOTAL		308	0.80 ± 0.03	13.44 ± 1.26	10.27 ± 0.79
*P*. *vivax*	East Sepik	Wosera	61	0.82 ± 0.03	11.20 ± 1.30	10.59 ± 1.19
	Madang	Malala	41	0.83 ± 0.02	11.00 ± 1.28	10.89 ± 1.29
	"	Mugil	54	0.84 ± 0.02	11.40 ± 1.00	10.99 ± 0.96
	"	Utu	37	0.83 ± 0.02	9.20 ± 0.95	9.19 ± 0.95
	TOTAL		193	0.84 ± 0.02	15.30 ± 1.87	11.99 ± 1.28

n = number of isolates genotyped after exclusion of outliers; *H*_*e*_ = expected heterozygosity; *A* = Mean number of alleles, *R*_*s*_ = Allelic richness.

### Effective population size

Using both the SMM and IAM models of evolution (see Materials and Methods), effective population size was estimated and found to be substantially greater for *P*. *vivax* than those for *P*. *falciparum* (Table 3). It must however be noted that in the absence of a microsatellite mutation rate for *P*. *vivax* (the same mutation rate was used for both species), and in light of the very large confidence intervals as a result of the variable estimates of the mutation rate, these results should be interpreted with care.

**Table 3:** Effective population size estimates for *P*. *falciparum* and *P*. *vivax* populations on the north coast of Papua New Guinea.

		SMM	IAM
*P*. *falciparum*	Wosera	10508 (4515–23936)	4387 (1885–9994)
	Malala	14696 (6315–33476)	5405 (2323–12313)
	Mugil	14017 (6023–31929)	5250 (2256–11960)
	Utu	6853 (2945–15612)	3329 (1431–7583)
	**Total**	**18871 (8109–42986)**	**6290 (2703–14328)**
*P*. *vivax*	Wosera	24108 (10360–54917)	7276 (3127–16573)
	Malala	25344 (10891–57732)	7492 (3220–17067)
	Mugil	31778 (13656–72388)	8547 (3673–19470)
	Utu	25883 (11123–58960)	7586 (3260–17279)
	**Total**	**30353 (13043–69142)**	**8323 (3577–18960)**

SMM = Stepwise mutation model, IAM = Infinite Alleles Model. The mutation rate of 1.59 X 10^−4^ for *P*. *falciparum* was used for both species. Numbers in brackets are lower and upper estimates derived from using the 95% confidence upper and lower mutation rates for *P*. *falciparum* (Lower = 6.98 X 10^−5^, Upper = 3.7 X 10^−4^) [11].

### Multilocus linkage disequilibrium

No evidence of multilocus LD was found in any of the *P*. *falciparum* catchments for all infections or single infections alone. Extremely low, yet significant LD was found when all catchments were combined (Table 4) however this was likely a result of subpopulation structure, a phenomenon known as the Wahlund effect [64]. For *P*. *vivax*, no significant LD was found with the exception of Wosera (Table 4) where two pairs of closely related haplotypes were found in one village (Nindigo). Linkage equilibrium was restored after removal of one of the shared nine loci haplotypes (*I* ^*S*^_*A*_ = 0.0052, *p* = 0.279). These closely-related isolates suggest instances of near clonal transmission or the presence of meiotic siblings among isolates from Nindigo [65].

**Table 4:** Estimates of multilocus linkage disequilibrium for *P*. *falciparum* and *P*. *vivax* populations on the north coast of Papua New Guinea.

		All Infections		Single Infections	
		n^a^	*I* ^*S*^_*A*_ (p-value)	n^a^	*I* ^*S*^_*A*_ (p-value)
*P*. *falciparum*	Wosera	38	-0.0049 (0.67)	32	-0.0076 (0.72)
	Malala	34	0.0015 (0.39)	22	0.0074 (0.71)
	Mugil	35	0.0013 (0.43)	21	0.0044 (0.38)
	Utu	52	0.0013 (0.42)	36	0.0033 (0.35)
	**TOTAL**	**159**	**0.0088 (0.01)**	**111**	**0.0046 (0.15)**
*P*. *vivax*	Wosera	53	0.0071 (0.05)	26	0.0146 (0.04)
	Malala	39	0.0079 (0.09)	18	0.0049 (0.32)
	Mugil	37	0.0066 (0.12)	10	-0.0235 (0.89)
	Utu	27	0.0093 (0.11)	9	-0.0018 (0.58)
	**TOTAL**	**156**	**0.0023 (0.08)**	**63**	**0.0053 (0.07)**

*I* ^*S*^_*A*_ = Index of Association,

^*a*^ = all infections,

^*b*^ = single infections only

### Population structure

**Fig 1:**
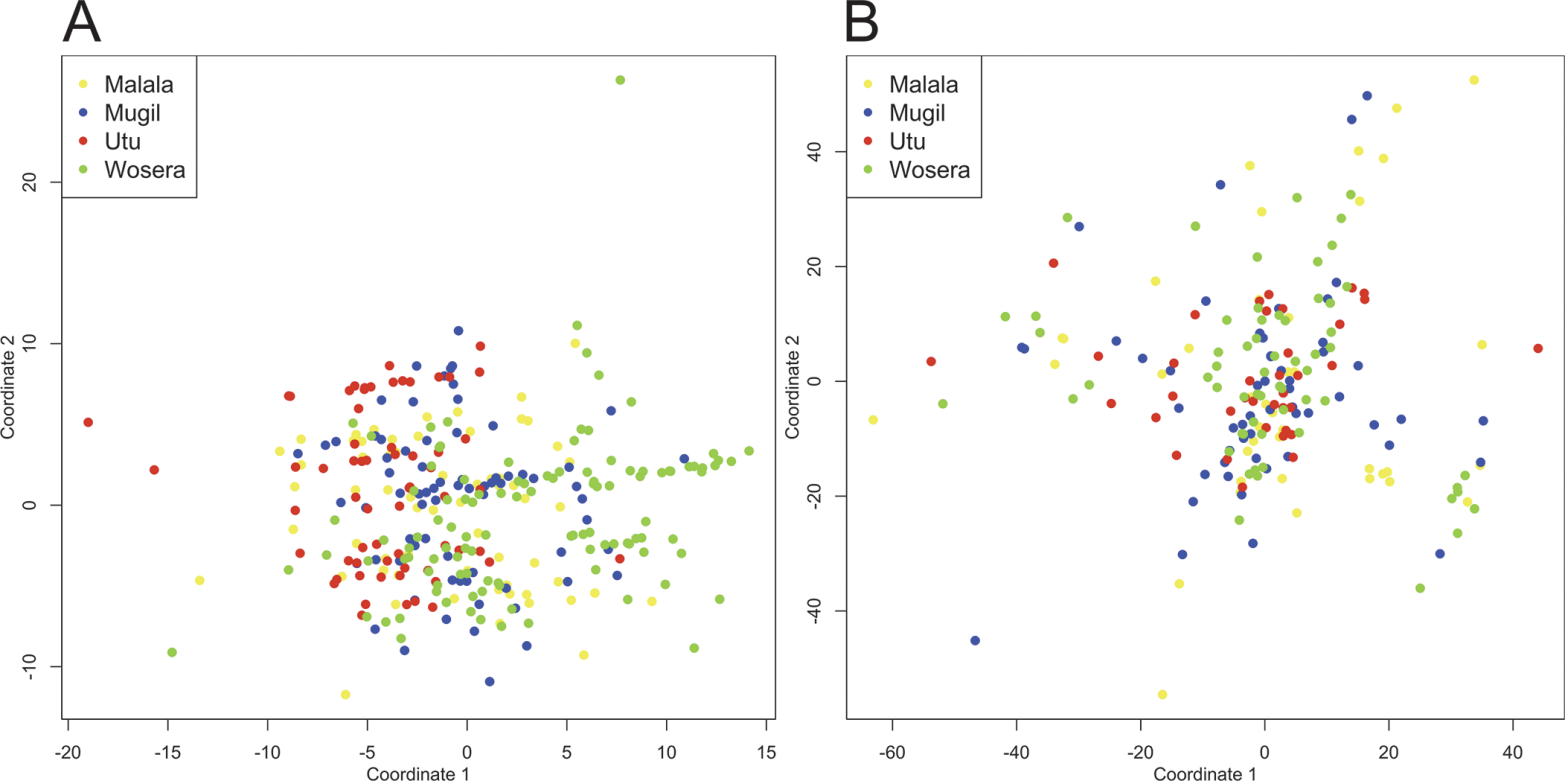
Multidimensional scaling analysis of *P*. *falciparum* and *P*. *vivax* microsatellite haplotypes from Papua New Guinea. Results of multidimensional scaling analysis (MDS) with cleaned datasets are shown for (A) *P*. *falciparum* and (B) *P*. *vivax*. Dots indicate individual microsatellite haplotypes and colours indicate the four sample catchment areas.

After exclusion of outlier samples and markers (see above), the MDS demonstrated clear divergence of *P*. *falciparum* populations in Madang province from the Wosera group of data points, indicating population structure at least between provinces (Fig 1A). In addition, Utu isolates were more closely clustered within the Madang cluster. For *P*. *vivax*, no such structure was visible in the MDS analysis, with isolates distributed throughout the main cluster independent of geographic origin (Fig 1B).

To measure levels of interpopulation differentiation between the different catchments, pairwise *G*_*ST*_ values were then determined. *G*_*ST*_ values were ten-fold higher between populations of *P*. *falciparum* (range of (0.0171, 0.0264)) than for those of *P*. *vivax* (range of (-0.0011, 0.0028), Table 5). In addition, the more robust measure Jost’s *D*, also showed higher levels of differentiation between *P*. *falciparum* populations (0.2105, 0.2811) than for *P*. *vivax* ((-0.0311, 0.0555),Table 5). *D* values can be interpreted as the mean proportion of pairwise private alleles between populations therefore for *P*. *falciparum*, 20–28% of alleles in each population are private, while for *P*. *vivax* less than 5% of alleles are private (S3 Table). The Mantel test for correlation between geographical and genetic distance found no significant correlation (S4 Table) indicating that the genetic differentiation observed was consistent with population fragmentation and genetic drift rather than isolation by distance.

**Table 5:** Estimates of genetic differentiation among *P*. *falciparum* and *P*. *vivax* populations on the North Coast of Papua New Guinea.

*P*. *falciparum*		*Wosera*	*Malala*	*Mugil*	*Utu*
	*Wosera*	-	0.0199	0.0255	0.0171
	*Malala*	0.2550	-	0.0192	0.0208
	*Mugil*	0.2811	0.2144	-	0.0264
	*Utu*	0.2105	0.2485	0.2560	-
*P*. *vivax*		Wosera	Malala	Mugil	Utu
	Wosera	-	0.0028	0.0015	0.0017
	Malala	0.0555	-	0.0023	0.0016
	Mugil	0.0264	0.0488	-	-0.0011
	Utu	0.0300	0.0415	-0.0311	-

Jost’s *D* values lower left, *G*_*ST*_ values upper right

**Fig 2:**
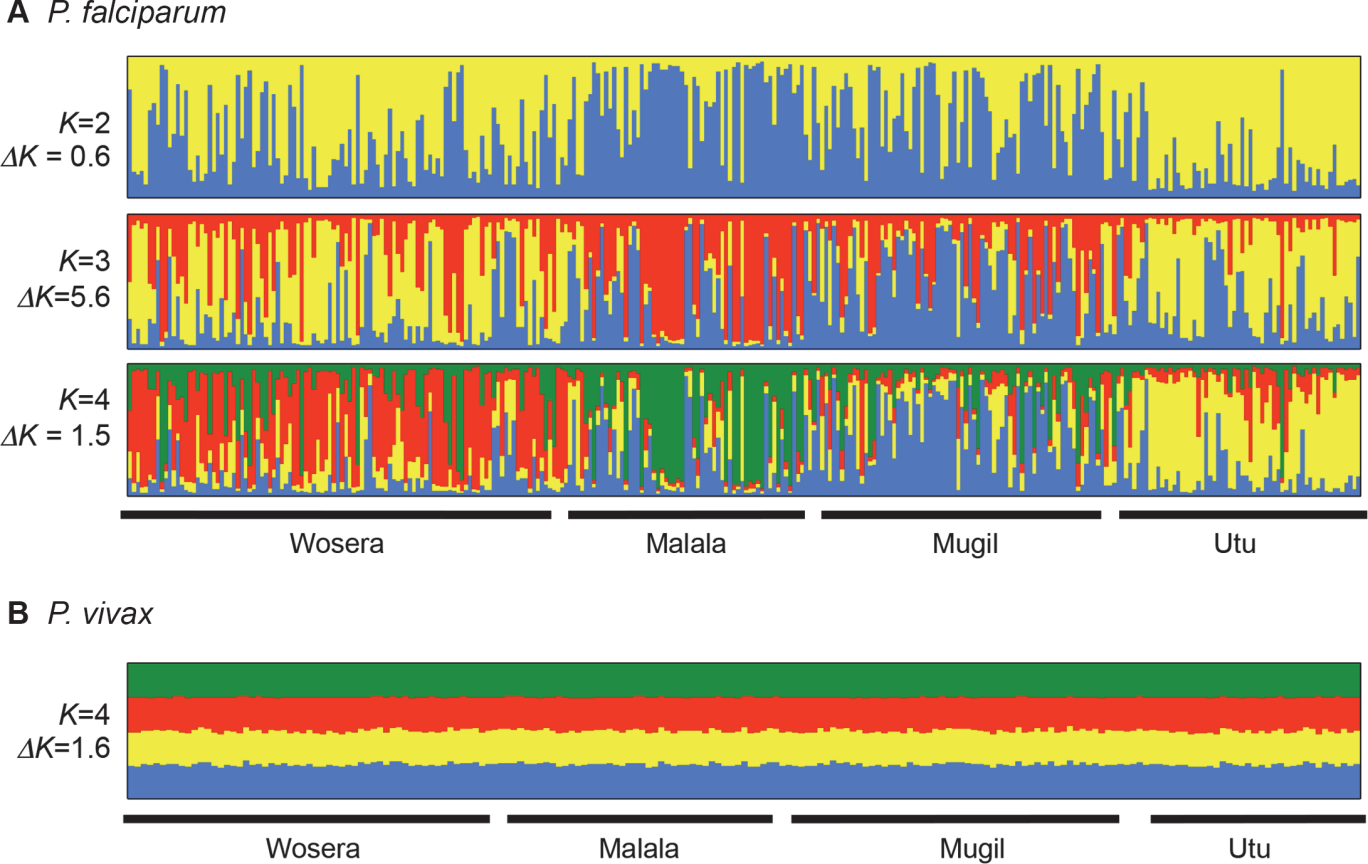
Bayesian cluster analysis of *P*. *falciparum* microsatellite haplotypes from Papua New Guinea. Individual ancestry coefficients are shown for (A) *P*. *falciparum* for *K* = 2–4 and (B) *P*. *vivax* for *K* = 4. Data generated in this study were analysed with *STRUCTURE* version 2.3.4 software [61]. Each vertical bar represents an individual haplotype and the membership coefficient (Q) within each of the genetic populations, as defined by the different colours. A chain length of 100,000 Monte Carlo Markov Chain iterations was used after a burn in of 10,000 steps using the admixture model and correlated allele frequencies. Each vertical bar represents an individual haplotype and its membership to each population is defined by the different colours. Black borders separate the four catchments.

In order to confirm the geospatial population structure observed, Bayesian cluster analysis of the haploid datasets was run before and after the exclusion of outliers and markers (TAA42 and Pv3.27) for both species using *STRUCTURE* version 2.3.4 [61]. Preliminary runs with 10,000 MCMC showed additional clustering by catchment in the *P*. *falciparum* dataset after MDS data cleaning (S4 Fig), however the use of a longer chain length of 100,000 MCMC also resolved four genetically distinct populations that were associated with each of the four catchments, with *ΔK* peaking at *K* = 3 (Fig 2A). Although there was evidence of some admixture among populations, this indicated three or four genetically distinct populations concordant with the catchment areas and the moderate values of genetic differentiation described above. For *P*. *vivax* there was no clear peak in the *ΔK* values. As the method of Evanno *et al*. is not reliable at identifying optimal *K* if *K* = 1 [62] we inspected the distribution of membership coefficients at *K* = 4. This partitioned all genotypes equally into four genetic clusters (Fig 2B), clearly demonstrating a complete lack of population structure. In addition we compared the new *P*. *vivax* data to that previously produced by Koepfli *et al*. [46] for nearby villages in East Sepik and Madang Provinces, as well as data from a village located in a remote inter-montane valley in the highlands (Sigimaru) and the Solomon Islands (S5 Fig). The results confirm a complete lack of detectable population structure for *P*. *vivax* in the SW Pacific region, based on the microsatellite markers [46].

## Discussion

Higher diversity among global *P*. *vivax* isolates than among *P*. *falciparum* isolates has been proposed to be consistent with more stable transmission over a long period of time and/or deeper evolutionary roots [66]. In some co-endemic areas, such as South America, the higher microsatellite diversity of *P*. *vivax* can be explained by its more stable transmission than *P*. *falciparum* [19,67]. A higher mutation rate of *P*. *vivax* microsatellites has also been proposed as one possible mechanism for the higher diversity of this species in South East Asia [17]. Within PNG, we have shown that despite comparably high transmission, as measured by EIR [31–33] and slightly lower infection prevalence than *P*. *falciparum* [36,37], *P*. *vivax* has greater genetic diversity and larger effective population sizes. Furthermore, we show for the first time that populations of *P*. *vivax* are highly admixed compared to sympatric populations of *P*. *falciparum*, which appear to be fragmented according to the analyses of genetic differentiation and population structure. In addition to the previous explanations for the higher diversity of *P*. *vivax*, we propose that the contrasting patterns of population structure at least partially reflect differences in the biology of these species. In particular, the ability of *P*. *vivax* to develop dormant hypnozoites and cause consecutive relapses is likely to provide more opportunities for the exchange and dissemination of alleles.

Higher genetic diversity suggests that *P*. *vivax* has greater evolutionary potential, which may allow it to adapt more rapidly to various environmental challenges including new antimalarial interventions. Indeed, our previous work has shown that *P*. *vivax* has much greater diversity in genes encoding the orthologs encoding the vaccine candidate AMA1, suggesting that it will be more difficult to vaccinate against all strains [68]. Because of its panmictic population structure in PNG, *P*. *vivax* may also be able to disseminate advantageous traits, such as drug resistance, more effectively than *P*. *falciparum*.

The generation of diversity in malaria parasite populations is facilitated by multiple clone infections [11], that permit the concurrent transmission of distinct clones which recombine in the mosquito midgut, generating novel genotypes [69]. Mean MOI values were similar between species, however the proportion of multiple infections differed considerably between species and between populations, possibly reflecting differences in transmission, which may be the result of relapsing *P*. *vivax* hypnozoites [70]. It should also be noted that the detection of clones would have been limited to a greater extent for *P*. *vivax* since it has lower density infections, in which case MOI for *P*. *vivax* may be underestimated. Whatever the case, the higher proportion of multiple infections in *P*. *vivax* provides a possible mechanism for generating and maintaining high levels of genetic diversity. That neither species showed any significant multilocus LD confirms high levels of outcrossing and overall high levels of transmission for both species, which contrasts with areas of low transmission where significant LD and clonal population structures have been observed in both species [17,19]. Differences in the proportion of multiple infections, if maintained over long periods of time, may be enough to drive the higher diversity of *P*. *vivax* compared to *P*. *falciparum* in PNG.

The contrasting patterns of population structure in PNG are consistent with *P*. *vivax* maintaining a large and relatively constant population size for a long period of time. For *P*. *falciparum*, the data suggest that allelic exchange between geographic regions may be or has been restricted or subject to population bottlenecks in the past. *P*. *vivax* was historically the dominant species in PNG [10,71] and the greater diversity observed could have resulted from an earlier colonisation of PNG by this species, and/or more frequent outcrossing as a result of a consistently higher proportion of multiple infections. While microsatellites can yield important information into population structure on an epidemiologically relevant time scale (tens to hundreds of years), studies using mitochondrial DNA (mtDNA) have provided great insight into the global spread and local population history of these two parasites. *P*. *vivax* has extraordinary mtDNA diversity in PNG with distinct haplotypes compared to other regions of the world. The most recent common ancestor has a wide age range (41–251 kya) and demographic modelling indicates a steady increase in population size over past millennia [72]. On the other hand, the *P*. *falciparum* population of PNG is dominated by one mtDNA haplotype also found in all other global *P*. *falciparum* populations, in addition to a number of rarer, private haplotypes, consistent with a relatively recent introduction and rapid population expansion within the last 30–50 kya [73,74]. It is therefore plausible that an earlier colonisation by *P*. *vivax* may have contributed to the higher baseline microsatellite diversity. It is also highly likely that recent events have shaped the population structure of *P*. *falciparum* and *P*. *vivax* in PNG, in a time frame detectable by microsatellite markers. The Indoor Residual Spraying programme, initiated in 1957 as part of the last Global Malaria Eradication Program, led to a substantial decline in the prevalence of all malaria species in PNG and particularly *P*. *falciparum*. After this control programme was abandoned in the late 1970s, malaria resurged with *P*. *falciparum* emerging as the dominant species [10,71], facilitated by the emergence of chloroquine resistance (CQR) [75]. The greater impact of control efforts and possibly CQR on *P*. *falciparum* is likely to have caused population bottlenecks, with consequent reductions in effective population size and limited gene flow leading to substantial genetic differentiation between populations. *P*. *vivax* cases also declined during this time, but as we have mentioned above, this parasite has high levels of population diversity even at low transmission [13,14,17,19,42,43], indicating that it may be less susceptible than *P*. *falciparum* to population bottlenecks resulting from declining transmission. In other geographic areas where interventions have succeeded in reducing the transmission of both species to very low levels, continuing malaria control efforts have had a less dramatic impact on *P*. *vivax*, suggesting that this parasite is more resistant to interventions [1,5,76]. This is likely to be a consequence of its unique biological characteristics, especially relapse, which provides more opportunities for outcrossing and dissemination of clones and may thus have allowed *P*. *vivax* to maintain large effective population sizes and gene flow even at low transmission.

In tropical areas such as PNG, relapsing hypnozoites contribute to at least 50% and up to 80% of all blood stage infections [70], with activation of hypnozoites allowing multiclonal infections even during times of low transmission intensity, in turn increasing the chances of outbreeding and reducing the likelihood of bottlenecks [77]. Dormancy may also aid the break down of population structure over large distances, as parasites hitchhike during human migration over greater distances than mosquitoes can travel. Human movement has been shown to be important for gene flow between island populations, however where human populations are continuous, gene flow likely occurs from a combination of both human and vector movement [78]. Hypnozoites not only facilitate the movement of parasite genotypes over large geographic distances, but constant reinfection and relapses of parasites from distinct inocula would allow for the recombination of distinct parasite clones and generations, promoting and maintaining high genetic diversity. In addition, *P*. *vivax* gametocytes appear in the blood stream earlier during an infection than *P*. *falciparum* gametocytes, and may be infective to mosquitoes before patients become symptomatic and seek treatment, thereby increasing the overall gene pool [6]. In the context of intensive malaria control, decreases in *P*. *vivax* diversity and subdivision of populations are thus less likely than for *P*. *falciparum*, and would only result from long-term, sustained interruption of transmission. This also provides an explanation for how *P*. *vivax* maintains high diversity even at low transmission and the absence of population structure observed here and in previous studies [46].

It is important to point out that the inclusion of outlier samples and markers in the population genetic analyses had a clear impact on the resolution of population structure. Clustering methods such as PCA and MDS are affected by outlier samples and markers, thus failure to remove these may hide true clustering patterns. These may be aberrant genotypes or infections imported from other endemic areas of PNG or beyond, however without samples from potential source populations this is not possible to confirm. After the removal of outliers, geographic population structure could be detected in *P*. *falciparum* samples based on one MDS coordinate. The bimodal clustering pattern observed along the other axis was driven by TAA42, which may be due to selection or technical artefacts. Running *STRUCTURE* for at least 100,000 MCMC steps also resolved the populations in the presence of TAA42, indicating that *STRUCTURE* was somewhat robust to outliers. However the analysis required more MCMC steps than are usually employed, indicating that the outliers produced a more complex likelihood, making it more difficult for the method to identify the maximum likelihood. As far as we are aware, data cleaning such as this is not routinely performed in microsatellite studies. Given the high probability of technical artefacts, and that population specific selection that may influence the allele frequencies of certain markers [63], we advocate the MDS and PCA data cleaning approaches for other studies of microsatellite-based population structure.

In conclusion, in an area of PNG where EIR and prevalence were comparably high at the time of sampling, *P*. *vivax* populations were consistently more genetically diverse, had larger effective population sizes and were more highly admixed compared to sympatric *P*. *falciparum* populations, which consisted of fragmented subpopulations. We propose this is driven by higher effective transmission of *P*. *vivax*, at least in part due to the re-activation of parasites from a pool of genetically diverse hypnozoites. The results underline the biological and historical differences between these two malaria species and illustrate why *P*. *vivax* is a greater challenge to elimination efforts. Distinct evolutionary histories [72–74,79], historically higher prevalence of *P*. *vivax* in PNG [80] and maintenance of high diversity at low transmission during the last eradication program are also likely to be contributing factors [10,37,46]. This warrants investigations to further elucidate the comparative demographic histories of *P*. *falciparum* and *P*. *vivax* in endemic areas, to understand the impact of past control efforts on the different species and to predict future outcomes of current control efforts. Since the samples were collected prior to intensified malaria control, these results will form the baseline against which future changes will be compared [81]. Significant reductions in the prevalence of both species in recent years, as the result of a nationwide control programme [1,82], may have had an impact upon these population structures. As intensive control has been maintained, reductions in diversity and increases in population structure could ultimately result, signalling the interruption of transmission [27]. The results also show that it will be important to measure changes in parasite population structure to inform control and elimination programs in areas where *P*. *vivax* is present, since traditional epidemiological parameters will underestimate true transmission rates.

## Supporting Information

S1 Fig Map of the study area. This map has been previously published in Schultz *et al*. 2010 *Malaria Journal* 2010, **9**:336 10.1186/1475-2875-9-336 (copyright A. E. Barry).
(DOCX)

S2 Fig Data cleaning for *P*. *falciparum* microsatellite haplotypes. Multidimensional Scaling (MDS) analyses for *P*. *falciparum*: (A) All genotyped samples. This analysis identified sample outliers that were distinct to the main cluster. Arrows indicate the five outliers that were apparent with coordinates one and two only. Removal and reanalysis of all coordinates revealed 12 outliers in total that were distinct to the main cluster. (B) After the removal of outlier samples. Separation of Wosera (green) and Madang (blue, yellow and red) was observed along the second principle component axis. Furthermore, the Utu (red) samples appeared to form a more compact cluster than other populations consistent with other analyses of genetic differentiation (Table 5) and population structure (Fig 1 and S4 Fig). The separation observed along the first component was unusual and was investigated further using Principle Components Analysis (PCA). (C) Biplot from PCA. PCA was only performed with individuals with no missing genotype data (N = 123). Inspection of the PCA biplot confirmed that the clustering was primarily driven by the marker TAA42, which has a bimodal allele frequency distribution. The cause of this is unknown and needs to be investigated. Further clustering was observable along the co-ordinate two axis with polyalpha showing some ability to discriminate between populations. As TAA42 did not conform to the patterns observed for other markers, genotypes for this locus were removed from the final dataset (see Fig 1A). Dots indicate individual microsatellite haplotypes and colours indicate the four sample catchment areas.
(TIF)

S3 Fig Datacleaning for *P*. *vivax* haplotypes. Multidimensional Scaling (MDS) analyses for *P*. *vivax* samples: (A) All genotyped samples. This identified sample outliers. Eight were found with these two coordinates as indicated by the arrows and samples in the ellipse. Removal and reanalysis of all coordinates revealed 11 outliers in total that were distinct to the main cluster. (B) After the removal of outlier samples. This shows samples from all populations were distributed throughout the main cluster demonstrating a lack of population structure as shown by other analyses (Fig 1 and Table 4). (C) Biplot for the PCA without the 11 outliers. PCA was only performed with individuals with no missing genotype data (N = 148). Inspection of the PCA biplot confirmed that some clustering was primarily driven by the marker Pv3.27, which has excess diversity in PNG populations [83]. As Pv3.27 did not conform to the patterns observed for other markers, genotypes for this locus were removed from the final dataset. Dots indicate individual microsatellite haplotypes and colours indicate the four sample catchment areas.
(TIF)

S4 Fig Bayesian cluster analysis of *P*. *falciparum* microsatellite haplotypes before and after data cleaning. Individual ancestry coefficients are shown for *P*. *falciparum* haplotypes (A) prior to data cleaning and (B) after data cleaning. Haplotypes were analysed with *Structure* version 2.3.4 software [61]. A chain length of 10,000 Monte Carlo Markov Chain iterations was used after a burn in of 10,000 using the admixture model and correlated allele frequencies. Results are shown for four populations (K = 4), demonstrating the partitioning of diversity amongst each of four genetic clusters. Each vertical bar represents an individual haplotype and the membership coefficient within each of the four genetic populations, as defined by the different colours. Black borders separate the four catchments. Note the greater definition of the four geographic populations following data cleaning.
(TIF)

S5 Fig Bayesian cluster analysis of *P*. *vivax* microsatellite haplotype data for Papua New Guinea and the Solomon Islands. Individual ancestry coefficients for *P*. *vivax* haplotypes from this study and previously published data. Haplotypes generated in this study were combined with those from Koepfli *et al*. [46] and analysed using Structure version 2.3.4 software [61]. A chain length of 100,000 Monte Carlo Markov Chain iterations was used after a burn in of 10,000 using the admixture model and correlated allele frequencies. Results are shown for two populations (K = 2). Each vertical bar represents an individual haplotype and the membership coefficient (Q) within each of the two genetic populations, as defined by the different colours. Black borders separate haplotypes from different catchments in the following order: East Sepik Province (Wosera, IlaitaA, IlaitaB, IlaitaC, Kunjingini, Ingambils, Kamanakor and Sunuhu), Madang Province (Malala, Mugil, Utu and Alexishafen), Sigimaru, which is a relatively isolated population located in an intermontane valley in the highlands, and the Solomon Islands (Guadalcanal).
(TIF)

S1 Table Estimates of genetic differentiation between single and dominant infection haplotype datasets for *P*. *falciparum* and *P*. *vivax*. *Jost’s D* values and *G*_ST_ were calculated between haplotypes reconstructed from single and dominant infections. All values were negative or very low indicating no genetic differentiation between single and dominant infection datasets. Therefore the two datasets were combined for each species and population thus increasing sample size.
(DOCX)

S2 Table Associations between different molecular epidemiological parameters. (DOCX)

S3 Table Microsatellite allele frequencies for *P*. *vivax* populations from Papua New Guinea. (DOCX)

S4 Table Associations between geographical and genetic distance (Mantel test). (DOCX)

S1 Text Strategy for exclusion of stutter peaks in fragment analysis. (DOCX)

S1 Dataset *P*. *falciparum* and *P*. *vivax* microsatellite haplotypes including all data and cleaned datasets. (XLSX)
