# Supplementary material for: Plasmodium vivax Populations Are More Genetically Diverse and Less Structured than Sympatric Plasmodium falciparum Populations
Source: PLoS Negl Trop Dis. 2015 Apr 15;9(4):e0003634. doi: 10.1371/journal.pntd.0003634 (PMC4398418; doi:10.1371/journal.pntd.0003634)
Supplement: S1 Fig — This map has been previously published in Schultz et al. 2010 Malaria Journal 2010, 9:336 10.1186/1475-2875-9-336 (copyright A. E. Barry). (DOCX) [file pntd.0003634.s001.docx]

**Figure S1 Map of the study area.**

This map has been previously published in Schultz *et al*. 2010 *Malaria Journal* 2010, **9**:336  doi:10.1186/1475-2875-9-336 (copyright A. E. Barry).

**
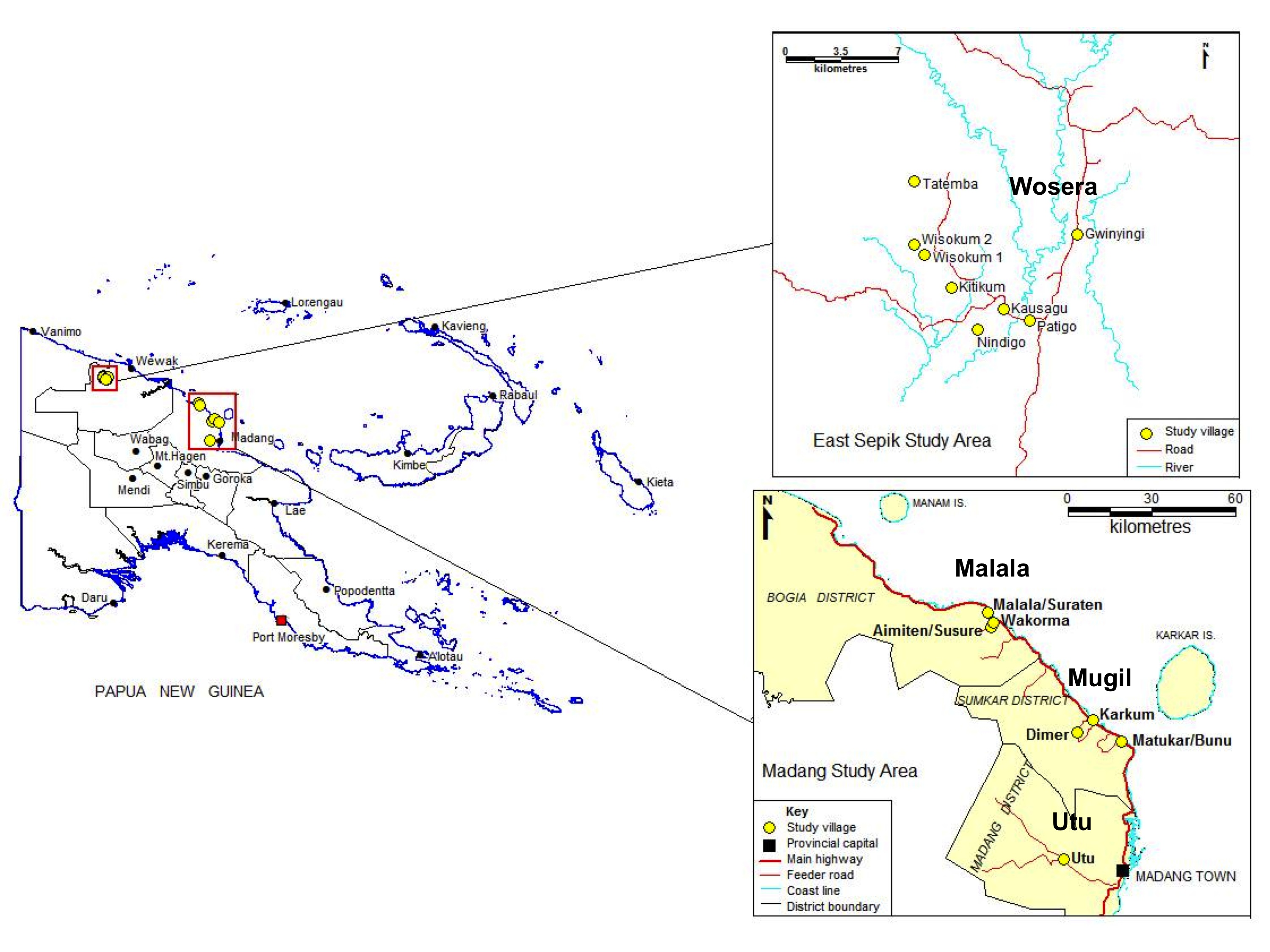
**
