# Supplementary figures and images for: Plasmodium vivax Populations Are More Genetically Diverse and Less Structured than Sympatric Plasmodium falciparum Populations
Source: PLoS Negl Trop Dis. 2015 Apr 15;9(4):e0003634. doi: 10.1371/journal.pntd.0003634 (PMC4398418; doi:10.1371/journal.pntd.0003634)

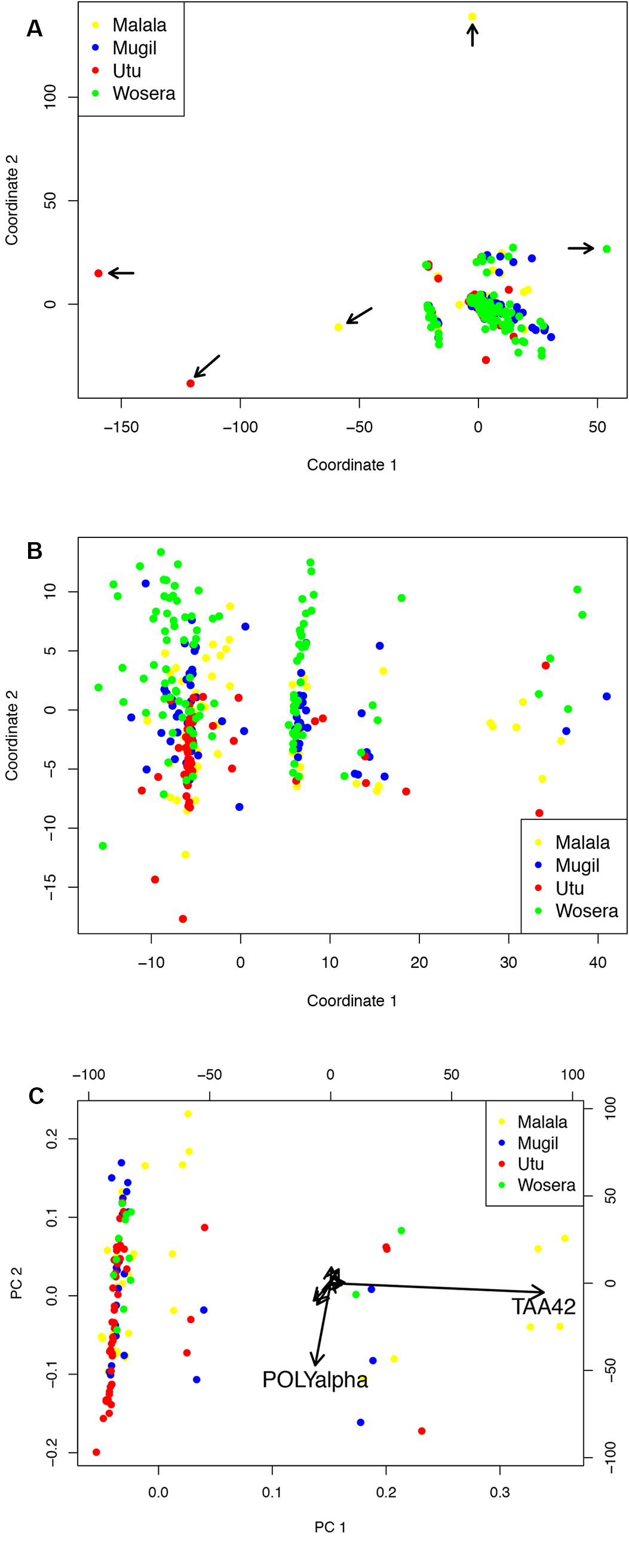

Supplement: S2 Fig — Multidimensional Scaling (MDS) analyses for P. falciparum: (A) All genotyped samples. This analysis identified sample outliers that were distinct to the main cluster. Arrows indicate the five outliers that were apparent with coordinates one and two only. Removal and reanalysis of all coordinates revealed 12 outliers in total that were distinct to the main cluster. (B) After the removal of outlier samples. Separation of Wosera (green) and Madang (blue, yellow and red) was observed along the second principle component axis. Furthermore, the Utu (red) samples appeared to form a more compact cluster than other populations consistent with other analyses of genetic differentiation (Table 5) and population structure (Fig 1 and S4 Fig). The separation observed along the first component was unusual and was investigated further using Principle Components Analysis (PCA). (C) Biplot from PCA. PCA was only performed with individuals with no missing genotype data (N = 123). Inspection of the PCA biplot confirmed that the clustering was primarily driven by the marker TAA42, which has a bimodal allele frequency distribution. The cause of this is unknown and needs to be investigated. Further clustering was observable along the co-ordinate two axis with polyalpha showing some ability to discriminate between populations. As TAA42 did not conform to the patterns observed for other markers, genotypes for this locus were removed from the final dataset (see Fig 1A). Dots indicate individual microsatellite haplotypes and colours indicate the four sample catchment areas. (TIF) [file pntd.0003634.s002.tif]

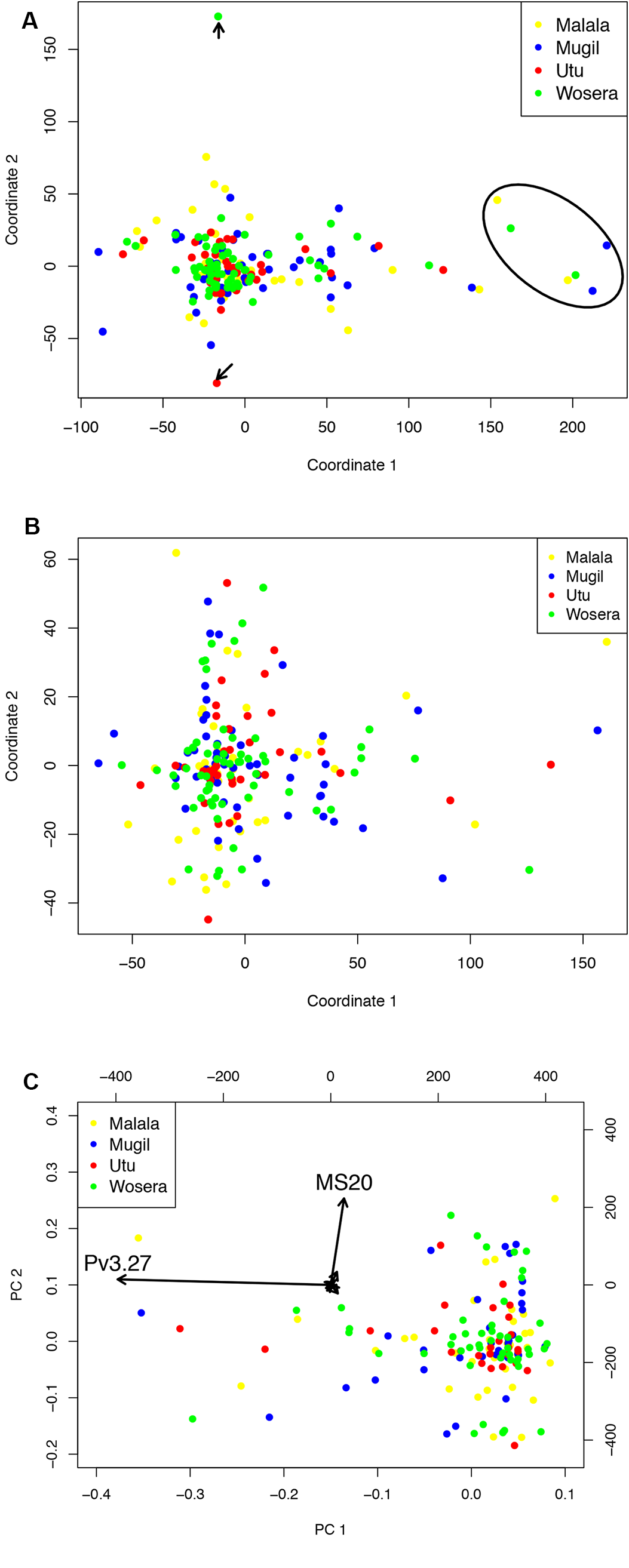

Supplement: S3 Fig — Multidimensional Scaling (MDS) analyses for P. vivax samples: (A) All genotyped samples. This identified sample outliers. Eight were found with these two coordinates as indicated by the arrows and samples in the ellipse. Removal and reanalysis of all coordinates revealed 11 outliers in total that were distinct to the main cluster. (B) After the removal of outlier samples. This shows samples from all populations were distributed throughout the main cluster demonstrating a lack of population structure as shown by other analyses (Fig 1 and Table 4). (C) Biplot for the PCA without the 11 outliers. PCA was only performed with individuals with no missing genotype data (N = 148). Inspection of the PCA biplot confirmed that some clustering was primarily driven by the marker Pv3.27, which has excess diversity in PNG populations [83]. As Pv3.27 did not conform to the patterns observed for other markers, genotypes for this locus were removed from the final dataset. Dots indicate individual microsatellite haplotypes and colours indicate the four sample catchment areas. (TIF) [file pntd.0003634.s003.tif]

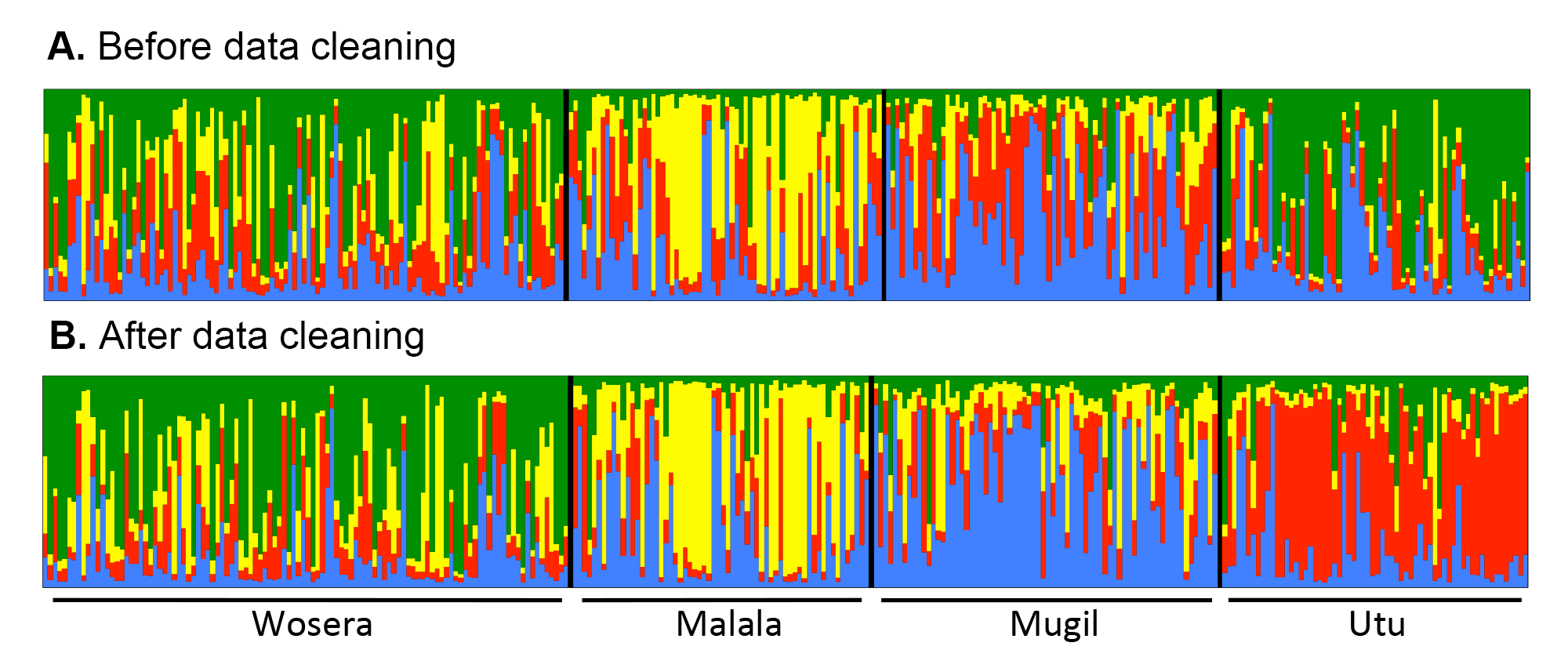

Supplement: S4 Fig — Individual ancestry coefficients are shown for P. falciparum haplotypes (A) prior to data cleaning and (B) after data cleaning. Haplotypes were analysed with Structure version 2.3.4 software [61]. A chain length of 10,000 Monte Carlo Markov Chain iterations was used after a burn in of 10,000 using the admixture model and correlated allele frequencies. Results are shown for four populations (K = 4), demonstrating the partitioning of diversity amongst each of four genetic clusters. Each vertical bar represents an individual haplotype and the membership coefficient within each of the four genetic populations, as defined by the different colours. Black borders separate the four catchments. Note the greater definition of the four geographic populations following data cleaning. (TIF) [file pntd.0003634.s004.tif]

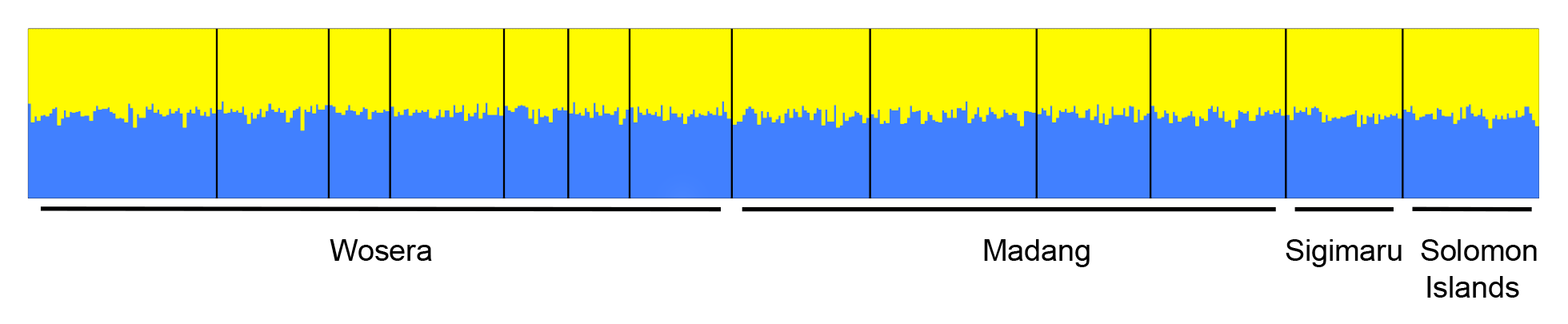

Supplement: S5 Fig — Individual ancestry coefficients for P. vivax haplotypes from this study and previously published data. Haplotypes generated in this study were combined with those from Koepfli et al. [46] and analysed using Structure version 2.3.4 software [61]. A chain length of 100,000 Monte Carlo Markov Chain iterations was used after a burn in of 10,000 using the admixture model and correlated allele frequencies. Results are shown for two populations (K = 2). Each vertical bar represents an individual haplotype and the membership coefficient (Q) within each of the two genetic populations, as defined by the different colours. Black borders separate haplotypes from different catchments in the following order: East Sepik Province (Wosera, IlaitaA, IlaitaB, IlaitaC, Kunjingini, Ingambils, Kamanakor and Sunuhu), Madang Province (Malala, Mugil, Utu and Alexishafen), Sigimaru, which is a relatively isolated population located in an intermontane valley in the highlands, and the Solomon Islands (Guadalcanal). (TIF) [file pntd.0003634.s005.tif]
