# Supplementary material for: Plasmodium vivax Populations Are More Genetically Diverse and Less Structured than Sympatric Plasmodium falciparum Populations
Source: PLoS Negl Trop Dis. 2015 Apr 15;9(4):e0003634. doi: 10.1371/journal.pntd.0003634 (PMC4398418; doi:10.1371/journal.pntd.0003634)
Supplement: S1 Table — Jost’s D values and G ST were calculated between haplotypes reconstructed from single and dominant infections. All values were negative or very low indicating no genetic differentiation between single and dominant infection datasets. Therefore the two datasets were combined for each species and population thus increasing sample size. (DOCX) [file pntd.0003634.s006.docx]

**Table S1. Estimates of genetic differentiation between single and dominant infection haplotype datasets for *P. falciparum* and *P. vivax*.**

Jost’s D values and G_ST_ were calculated between haplotypes reconstructed from single and dominant infections. All values were negative or very low indicating no genetic differentiation between single and dominant infection datasets. Therefore the two datasets were combined for each species and population thus increasing sample size.

|  | *P. falciparum* | | *P. vivax* | |
| --- | --- | --- | --- | --- |
|  | *D* | *G_ST_* | *D* | *G_ST_* |
| Wosera | -0.0143 | -0.0005 | -0.0109 | 9.3090 x 10^-05^ |
| Malala | -0.0093 | 0.0001 | -0.0604 | -0.0023 |
| Mugil | -0.0519 | -0.0043 | -0.0300 | -0.0018 |
| Utu | -0.0481 | -0.0034 | -0.1109 | -0.0049 |
