# Supplementary material for: Plasmodium vivax Populations Are More Genetically Diverse and Less Structured than Sympatric Plasmodium falciparum Populations
Source: PLoS Negl Trop Dis. 2015 Apr 15;9(4):e0003634. doi: 10.1371/journal.pntd.0003634 (PMC4398418; doi:10.1371/journal.pntd.0003634)
Supplement: S2 Table — (DOCX) [file pntd.0003634.s007.docx]

**Table S2.** **Associations between different molecular epidemiological parameters**

| *P.falciparum* |  | Prevalence (%) | Mean  MOI | Polyclonal (%) | *H*_e_ | *R*_s_ |
| --- | --- | --- | --- | --- | --- | --- |
|  | Prevalence |  | 0.42 | 1.08 | 0.75 | 0.33 |
|  | MOI | 0.60 |  | 0.33 | 0.75 | 1.08 |
|  | Polyclonal | 0 | -0.80 |  | 0.92 | 0.42 |
|  | *H*_e_ | -0.40 | -0.40 | 0.20 |  | 0.92 |
|  | *R*_s_ | -0.80 | 0 | -0.60 | 0.20 |  |
|  |  |  |  |  |  |  |
| *P. vivax* |  | Prevalence (%) | Mean MOI | Polyclonal (%) | *H*_e_ | *R*_s_ |
|  | Prevalence |  | 0.5 | 0.33 | 0.5 | 0.33 |
|  | MOI | 0.63 |  | 0.5 | 0.17 | 1 |
|  | Polyclonal | -0.80 | -0.32 |  | 0.5 | 0.08 |
|  | *H*_e_ | 0.63 | 1 | -0.32 |  | 1 |
|  | *R_s_* | 0.80 | 0.32 | -1 | 0.32 |  |

Spearman’s correlation coefficients are shown in the lower diagnonal of the matrix, *p*-values are shown on the upper diagonal. MOI = multiplicity of infection, *H*_e_ = Expected heterozygosity, *R*_s_ = allelic richness.
