# Supplementary material for: Plasmodium vivax Populations Are More Genetically Diverse and Less Structured than Sympatric Plasmodium falciparum Populations
Source: PLoS Negl Trop Dis. 2015 Apr 15;9(4):e0003634. doi: 10.1371/journal.pntd.0003634 (PMC4398418; doi:10.1371/journal.pntd.0003634)
Supplement: S3 Table — (DOCX) [file pntd.0003634.s008.docx]

**Table S3.** Microsatellite allele frequencies for *Plasmodium vivax* populations from Papua New Guinea, after the exclusion of outliers.

|  | **Allele** | **Fragment length** | **Malala** | **Mugil** | **Utu** | **Wosera** | **Overall** |
| --- | --- | --- | --- | --- | --- | --- | --- |
| MS1 | 1 | 223 | 0.05 |  |  |  | 0.01 |
|  | 2 | 226 | 0.03 |  | 0.06 | 0.02 | 0.02 |
|  | 3 | 229 | 0.15 | 0.14 | 0.19 | 0.25 | 0.19 |
|  | 4 | 232 | 0.43 | 0.37 | 0.42 | 0.30 | 0.37 |
|  | 5 | 235 | 0.30 | 0.33 | 0.28 | 0.36 | 0.32 |
|  | 6 | 238 | 0.03 | 0.10 |  | 0.08 | 0.06 |
|  | 7 | 241 | 0.03 | 0.04 | 0.03 |  | 0.02 |
|  | 8 | 244 |  | 0.02 | 0.03 |  | 0.01 |
|  | *No. samples* |  | *40* | *51* | *36* | *61* | *188* |
| MS2 | 1 | 169 | 0.03 | 0.02 |  |  | 0.01 |
|  | 2 | 181 | 0.03 |  |  |  | 0.01 |
|  | 3 | 201 |  | 0.02 |  |  | 0.01 |
|  | 4 | 205 | 0.03 |  |  | 0.04 | 0.02 |
|  | 5 | 209 | 0.05 | 0.08 | 0.06 |  | 0.04 |
|  | 6 | 213 | 0.05 | 0.08 | 0.09 | 0.11 | 0.08 |
|  | 7 | 217 | 0.13 | 0.02 | 0.06 | 0.11 | 0.08 |
|  | 8 | 221 | 0.23 | 0.16 | 0.15 | 0.11 | 0.16 |
|  | 9 | 225 | 0.08 | 0.24 | 0.21 | 0.11 | 0.16 |
|  | 10 | 229 | 0.08 | 0.10 | 0.09 | 0.19 | 0.12 |
|  | 11 | 233 | 0.05 | 0.06 | 0.03 | 0.05 | 0.05 |
|  | 12 | 237 | 0.03 | 0.06 | 0.06 | 0.05 | 0.05 |
|  | 13 | 241 | 0.05 | 0.04 | 0.15 | 0.07 | 0.07 |
|  | 14 | 245 |  | 0.02 |  | 0.07 | 0.03 |
|  | 15 | 249 |  | 0.02 | 0.12 | 0.02 | 0.03 |
|  | 16 | 253 | 0.03 |  |  | 0.05 | 0.02 |
|  | 17 | 257 | 0.05 | 0.04 |  | 0.02 | 0.03 |
|  | 18 | 265 | 0.03 |  |  | 0.02 | 0.01 |
|  | 19 | 273 | 0.03 |  |  |  | 0.01 |
|  | 20 | 277 |  | 0.02 |  |  | 0.01 |
|  | 21 | 285 | 0.05 |  |  |  | 0.01 |
|  | *No. samples* |  | *39* | *49* | *34* | *57* | *179* |
| MS5 | 1 | 161 | 0.03 | 0.04 |  | 0.02 | 0.02 |
|  | 2 | 164 | 0.05 |  |  | 0.05 | 0.03 |
|  | 3 | 167 |  |  | 0.03 |  | 0.01 |
|  | 4 | 170 | 0.03 | 0.02 | 0.03 | 0.07 | 0.04 |
|  | 5 | 173 | 0.19 | 0.09 | 0.15 | 0.10 | 0.13 |
|  | 6 | 176 | 0.14 | 0.19 | 0.06 | 0.19 | 0.15 |
|  | 7 | 179 | 0.14 | 0.19 | 0.15 | 0.19 | 0.17 |
|  | 8 | 182 | 0.22 | 0.17 | 0.24 | 0.07 | 0.16 |
|  | 9 | 185 | 0.11 | 0.04 | 0.12 | 0.10 | 0.09 |
|  | 10 | 188 |  | 0.08 |  | 0.09 | 0.05 |
|  | 11 | 191 | 0.03 | 0.08 | 0.12 |  | 0.05 |
|  | 12 | 194 | 0.03 | 0.04 | 0.03 | 0.02 | 0.03 |
|  | 13 | 197 | 0.03 | 0.02 | 0.06 | 0.05 | 0.04 |
|  | 14 | 200 |  | 0.04 |  | 0.02 | 0.02 |
|  | 15 | 203 | 0.03 |  |  | 0.02 | 0.01 |
|  | 16 | 206 |  |  |  | 0.02 | 0.01 |
|  | 17 | 209 |  | 0.02 |  |  | 0.01 |
|  | *No. samples* |  | *37* | *53* | *33* | *58* | *181* |
| MS6 | 1 | 213 | 0.10 | 0.08 | 0.08 | 0.09 | 0.09 |
|  | 2 | 222 | 0.02 | 0.02 |  | 0.02 | 0.02 |
|  | 3 | 225 | 0.02 |  |  | 0.02 | 0.01 |
|  | 4 | 228 | 0.02 | 0.04 |  |  | 0.02 |
|  | 5 | 234 | 0.07 | 0.04 |  | 0.03 | 0.04 |
|  | 6 | 237 | 0.02 |  | 0.05 | 0.05 | 0.03 |
|  | 7 | 240 |  | 0.04 | 0.08 | 0.03 | 0.04 |
|  | 8 | 243 | 0.05 | 0.04 | 0.08 | 0.05 | 0.05 |
|  | 9 | 246 | 0.10 | 0.20 | 0.27 | 0.19 | 0.19 |
|  | 10 | 249 | 0.29 | 0.22 | 0.24 | 0.14 | 0.21 |
|  | 11 | 252 | 0.17 | 0.16 | 0.03 | 0.10 | 0.12 |
|  | 12 | 255 | 0.10 | 0.04 | 0.11 | 0.14 | 0.10 |
|  | 13 | 258 | 0.02 | 0.10 | 0.03 | 0.05 | 0.05 |
|  | 14 | 261 |  | 0.02 |  | 0.07 | 0.03 |
|  | 15 | 264 |  | 0.02 | 0.03 | 0.02 | 0.02 |
|  | *No. samples* |  | *41* | *51* | *37* | *58* | *187* |
| MS7 | 1 | 140 |  | 0.02 |  |  | 0.01 |
|  | 2 | 143 | 0.05 | 0.02 | 0.06 | 0.02 | 0.03 |
|  | 3 | 146 | 0.13 | 0.20 | 0.34 | 0.19 | 0.21 |
|  | 4 | 149 | 0.33 | 0.16 | 0.26 | 0.41 | 0.29 |
|  | 5 | 152 | 0.25 | 0.24 | 0.17 | 0.20 | 0.22 |
|  | 6 | 155 | 0.13 | 0.08 | 0.03 | 0.10 | 0.09 |
|  | 7 | 158 | 0.05 | 0.18 | 0.09 | 0.05 | 0.09 |
|  | 8 | 161 | 0.03 | 0.04 |  | 0.03 | 0.03 |
|  | 9 | 164 |  | 0.04 |  |  | 0.01 |
|  | 10 | 167 | 0.03 | 0.02 | 0.03 |  | 0.02 |
|  | 11 | 173 | 0.03 |  | 0.03 |  | 0.01 |
|  | 12 | 227 |  | 0.02 |  |  | 0.01 |
|  | *No. samples* |  | *40* | *51* | *35* | *59* | *185* |
| MS9 | 1 | 146 |  |  | 0.03 |  | 0.01 |
|  | 2 | 152 | 0.03 | 0.07 |  |  | 0.03 |
|  | 3 | 155 | 0.15 | 0.06 | 0.18 | 0.13 | 0.12 |
|  | 4 | 158 | 0.05 | 0.24 | 0.21 | 0.43 | 0.26 |
|  | 5 | 161 | 0.31 | 0.20 | 0.26 | 0.15 | 0.22 |
|  | 6 | 164 | 0.18 | 0.20 | 0.21 | 0.16 | 0.19 |
|  | 7 | 167 | 0.05 | 0.06 | 0.12 | 0.03 | 0.06 |
|  | 8 | 170 | 0.08 | 0.06 |  | 0.07 | 0.05 |
|  | 9 | 173 | 0.13 | 0.07 |  | 0.03 | 0.06 |
|  | 10 | 176 | 0.03 | 0.02 |  |  | 0.01 |
|  | 11 | 179 |  | 0.02 |  |  | 0.01 |
|  | *No. samples* |  | *39* | *54* | *34* | *61* | *188* |
| MS10 | 1 | 163 | 0.03 |  |  | 0.02 | 0.01 |
|  | 2 | 166 |  |  | 0.06 |  | 0.01 |
|  | 3 | 169 |  | 0.02 |  | 0.05 | 0.02 |
|  | 4 | 172 |  | 0.08 | 0.06 | 0.02 | 0.04 |
|  | 5 | 175 | 0.05 | 0.02 |  | 0.07 | 0.04 |
|  | 6 | 178 | 0.03 | 0.08 | 0.06 | 0.05 | 0.06 |
|  | 7 | 181 | 0.16 | 0.14 | 0.26 | 0.08 | 0.15 |
|  | 8 | 184 | 0.14 | 0.10 | 0.14 | 0.17 | 0.14 |
|  | 9 | 187 |  | 0.14 |  | 0.02 | 0.04 |
|  | 10 | 190 | 0.14 |  | 0.03 | 0.10 | 0.07 |
|  | 11 | 193 | 0.05 | 0.08 | 0.09 | 0.10 | 0.08 |
|  | 12 | 196 | 0.30 | 0.20 | 0.23 | 0.22 | 0.23 |
|  | 13 | 199 |  | 0.08 |  | 0.02 | 0.03 |
|  | 14 | 202 |  | 0.02 |  | 0.02 | 0.01 |
|  | 15 | 205 | 0.08 | 0.02 | 0.03 | 0.07 | 0.05 |
|  | 16 | 208 |  |  |  | 0.02 | 0.01 |
|  | 17 | 211 | 0.03 |  | 0.03 |  | 0.01 |
|  | 18 | 214 |  |  | 0.03 |  | 0.01 |
|  | *No. samples* |  | *37* | *49* | *35* | *60* | *181* |
| MS12 | 1 | 186 |  | 0.02 |  | 0.02 | 0.01 |
|  | 2 | 207 | 0.15 | 0.06 | 0.03 | 0.04 | 0.07 |
|  | 3 | 210 | 0.44 | 0.33 | 0.29 | 0.51 | 0.40 |
|  | 4 | 213 | 0.21 | 0.38 | 0.40 | 0.30 | 0.32 |
|  | 5 | 216 | 0.15 | 0.15 | 0.17 | 0.07 | 0.13 |
|  | 6 | 219 | 0.05 | 0.02 | 0.11 | 0.05 | 0.06 |
|  | 7 | 222 |  |  |  | 0.02 | 0.01 |
|  | 8 | 228 |  | 0.04 |  |  | 0.01 |
|  | *No. samples* |  | *39* | *48* | *35* | *57* | *179* |
| MS15 | 1 | 237 | 0.03 |  |  |  | 0.01 |
|  | 2 | 240 | 0.03 |  | 0.06 | 0.02 | 0.02 |
|  | 3 | 243 | 0.13 | 0.06 | 0.12 | 0.05 | 0.08 |
|  | 4 | 246 |  | 0.08 | 0.12 | 0.17 | 0.10 |
|  | 5 | 249 | 0.33 | 0.14 | 0.18 | 0.23 | 0.22 |
|  | 6 | 252 | 0.18 | 0.16 | 0.06 | 0.07 | 0.11 |
|  | 7 | 255 | 0.03 | 0.12 | 0.09 | 0.15 | 0.10 |
|  | 8 | 258 | 0.18 | 0.12 | 0.15 | 0.15 | 0.15 |
|  | 9 | 261 | 0.05 | 0.22 | 0.15 | 0.02 | 0.10 |
|  | 10 | 264 | 0.03 | 0.02 | 0.03 | 0.03 | 0.03 |
|  | 11 | 267 |  |  |  | 0.03 | 0.01 |
|  | 12 | 270 | 0.03 | 0.02 |  | 0.02 | 0.02 |
|  | 13 | 273 |  |  |  | 0.03 | 0.01 |
|  | 14 | 288 |  | 0.02 | 0.03 |  | 0.01 |
|  | 15 | 291 | 0.03 | 0.02 |  | 0.02 | 0.02 |
|  | 16 | 294 |  |  | 0.03 | 0.02 | 0.01 |
|  | *No. samples* |  | *40* | *49* | *34* | *60* | *183* |
| MS20 | 1 | 158 |  |  |  | 0.02 | 0.01 |
|  | 2 | 161 | 0.05 | 0.07 | 0.03 | 0.10 | 0.07 |
|  | 3 | 173 | 0.03 |  |  |  | 0.01 |
|  | 4 | 176 | 0.08 | 0.04 |  |  | 0.03 |
|  | 5 | 179 | 0.03 | 0.02 |  |  | 0.01 |
|  | 6 | 182 | 0.03 |  |  |  | 0.01 |
|  | 7 | 185 | 0.03 |  | 0.06 |  | 0.02 |
|  | 8 | 188 | 0.05 | 0.04 | 0.03 | 0.03 | 0.04 |
|  | 9 | 191 | 0.03 | 0.13 | 0.11 | 0.07 | 0.08 |
|  | 10 | 194 | 0.08 | 0.20 | 0.23 | 0.16 | 0.16 |
|  | 11 | 197 | 0.30 | 0.15 | 0.09 | 0.19 | 0.18 |
|  | 12 | 200 | 0.08 | 0.13 | 0.14 | 0.16 | 0.13 |
|  | 13 | 203 | 0.03 |  | 0.06 | 0.05 | 0.03 |
|  | 14 | 206 |  |  |  | 0.03 | 0.01 |
|  | 15 | 209 | 0.03 | 0.02 | 0.03 | 0.05 | 0.03 |
|  | 16 | 212 | 0.08 | 0.02 | 0.09 |  | 0.04 |
|  | 17 | 215 |  | 0.02 | 0.03 | 0.02 | 0.02 |
|  | 18 | 218 | 0.03 | 0.04 |  |  | 0.02 |
|  | 19 | 221 |  |  | 0.03 |  | 0.01 |
|  | 20 | 224 |  | 0.02 | 0.03 | 0.03 | 0.02 |
|  | 21 | 227 | 0.05 |  |  |  | 0.01 |
|  | 22 | 230 |  | 0.02 |  |  | 0.01 |
|  | 23 | 233 | 0.03 | 0.02 |  |  | 0.01 |
|  | 24 | 236 |  | 0.04 | 0.03 | 0.03 | 0.03 |
|  | 25 | 239 |  |  |  | 0.02 | 0.01 |
|  | 26 | 242 |  |  | 0.03 | 0.03 | 0.02 |
|  | 27 | 251 | 0.03 |  |  |  | 0.01 |
|  | *No. samples* |  | *40* | *46* | *35* | *58* | *179* |
|  | **Total samples** |  | **41** | **54** | **37** | **61** | **193** |
