# Supplementary material for: Plasmodium vivax Populations Are More Genetically Diverse and Less Structured than Sympatric Plasmodium falciparum Populations
Source: PLoS Negl Trop Dis. 2015 Apr 15;9(4):e0003634. doi: 10.1371/journal.pntd.0003634 (PMC4398418; doi:10.1371/journal.pntd.0003634)
Supplement: S4 Table — (DOCX) [file pntd.0003634.s009.docx]

**Table S4.** **Associations between geographical and genetic distance (Mantel test).**

|  | *P. falciparum* | *P. vivax* |
| --- | --- | --- |
| km : *D* | 0.162 (0.365) | 0.168 (0.415) |
| km : *G_ST_* | -0.212 (0.692) | 0.362 (0.504) |

Values in brackets are *p*-values
