# Supplementary material for: Plasmodium vivax Populations Are More Genetically Diverse and Less Structured than Sympatric Plasmodium falciparum Populations
Source: PLoS Negl Trop Dis. 2015 Apr 15;9(4):e0003634. doi: 10.1371/journal.pntd.0003634 (PMC4398418; doi:10.1371/journal.pntd.0003634)
Supplement: S1 Text — (DOCX) [file pntd.0003634.s010.docx]

**Text S1. Strategy for exclusion of stutter peaks in fragment analysis.**

For electropherogram analysis in Genemapper version 4.0, the minimum fluorescence was set to 500 Random Fluorescence Units (RFU) for all colours except orange, the colour of the size standard, which was set to 200RFU as this reduced the amount of manual checking required for the size standard. Stutter window was set to 3.5 for 3bp repeats and 4.5 for 4bp repeats. The stutter ratio was set to 0.4 for all markers except MS16, which displayed far greater stuttering and was consequently set to 0.6. The stutter detection only applied to shorter alleles, with longer alleles within the stutter window subject to the standard 30% cut-off threshold because stutter peaks longer than the fragments from which they are derived were uncommon. Samples with low fluorescence were reanalysed with a minimum fluorescence of 100 RFU.
